# The Work of Worms

**Published:** 1882-01

**Authors:** Geo. J. Romanes


### POPULAR SCIENCE DEPARTMENT.

### THE WORK OF WORMS.

If the world were not already accustomed to the unprecedented fertility of Mr. Darwin's genius, it might well be disposed to marvel at the appearance of yet another work, now added to the magnificent array of those which bear his name. But feelings of wonder at Mr. Darwin's activity have long ago been sated, and most of us have grown to regard his powers of research as belonging to a class *sui generis*, to which the ordinary measures of working capacity do not apply. Be our feelings of wonder, however, what they may, it is most gratifying to find that this latest work from the hand of our illustrious countryman is in every way worthy of its predecessors. Everywhere throughout the book we meet with the distinctive attributes of Mr. Darwin's mind. Beginning with matters of the most common knowledge, which at first sight appear to furnish the most unpromising material, he proceeds by close observation of details and sagacious manipulation of facts to establish general truths of the most far-reaching importance in directions where we should least have expected any such truths to lie.

But to avoid the presumption of seeming to commend the work of so great a master, we shall proceed at once to render an epitome of the work itself. This, as its title is sufficient to denote, is an extension of the celebrated paper, "On the Formation of Mould," read before the Geological Society in 1837 (See Trans. Geol. Soc., vol. v., p. 505); but the extension is so considerable that the present volume is really a new work. The subject, of course, is the same; but the later observations, while tending to confirm, and in fact to demonstrate, the conclusions based upon the former, have served to swell a short paper into a book of over 300 pages. Alluding to this paper, Mr. Darwin writes:

"It was there shown that small fragments of burnt marl, cinders, etc., which had been thickly strewed over the surface of several meadows, were found after a few years lying at a depth of some inches beneath the turf, but still forming a layer. This apparent sinking of superficial bodies is due, as was first suggested to me by Mr. Wedgwood of Maer Hall, in Staffordshire, to the large quantity of fine earth continually brought up to the surface by worms in the form of castings. These castings are sooner or later spread out, and cover up any object left on the surface. I was thus led to conclude that all the vegetable mould over the whole country has passed many times through the intestinal canal of worms. Hence the term 'animal mould' would be more appropriate than that commonly used of 'vegetable mould.'"

Dealing next with criticisms which from time to time have been made upon his original paper, Mr. Darwin quotes one from Mr. Fish, which we may here requote on account of its instructive character. "Considering their weakness and their size, the work they are represented to have accomplished is stupendous." On which Mr. Darwin observes:

"Here we have an instance of that inability to sum up the effects of a continually recurring cause which has often retarded the progress of science, as formerly in the case of geology, and more recently in that of the principle of evolution." He then adds :

"Although these several objections seemed to me to have no weight, yet I resolved to make more observations of the same kind as those published, and to attack the problem on another side : namely, to weigh all the casting thrown up within a given time in a measured space, instead of ascertaining the rate at which objects left on the surface were buried by worms. But some of my observations have been rendered almost superfluous by an admirable paper by Von Hensen, already alluded to, which appeared in 1877. Before entering on details with respect to the castings, it will be advisable to give some account of the habits of worms from my own observations and from those of other naturalists."

Of these habits, the most interesting are as follows :

Although earth-worms are, properly speaking, terrestrial animals, they are still "like the other members of the great class of annelids to which they belong," semi-aquatic. For while dry air is quickly fatal to them, they may live when completely submerged in water for nearly four months. Normally they live in burrows, and generally lie motionless just at the mouth of the latter, so that by looking down into the burrows, the heads of the worms can be seen. This habit of lying near the surface leads to their destruction in enormous numbers by birds. For,

"Every morning during certain seasons of the year, the thrushes and blackbirds on all the lawns throughout the country draw out of their holes an astonishing number of worms ; and this they could not do unless they lay close to the surface. It is not probable that worms behave in this manner for the sake of breathing fresh air, for we have seen that they can live a long time under water. I believe that they lie near the surface for the sake of warmth, especially in the morning ; and we shall hereafter find that they often coat the mouths of their burrows with leaves, apparently to prevent their bodies from coming into close contact with the cold damp earth. It is said that they completely close their burrows during the winter."

As regards powers of special sense, it has been observed by Hoffmeister that, although destitute of eyes, earth worms are sensitive to light, time, however, being required for the summation of the stimulus before it is responded to. It is only the anterior extremity of the body, where the cerebral ganglia are situated, that is thus sensitive to light.

These observations have been confirmed by Mr. Darwin. He further found that the color of the light apparently made no difference in the result, nor did partly filtering out the heat rays by means of a sheet of glass; while a dull red heated poker, held at such a distance from the worms as would cause a sensible degree of warmth to the hand, did not disturb them nearly so much as the light from a candle concentrated by a lens. The sensitiveness to light is less when a worm is engaged in eating or in dragging leaves into its burrow, a fact which Mr. Darwin is disposed to consider analogous to what in higher animals we know as the distracting influence of attention. When not engaged in any active operation, the sensitiveness of worms to light is so considerable that "when a worm is suddenly illuminated it dashes like a rabbit into its burrow."

With respect to hearing, all the experiments went to show that worms are totally deaf to all kinds of aerial vibration, although extremely sensitive to the vibration of any solid object with which they may be in contact, as was shown, among other ways, by placing flowerpots containing worms in their burrows upon a piano; on striking single notes, whether high or low, the worms instantly retreated. In this connection, also, the following may be quoted:

"It has often been said that if the ground is beaten or otherwise made to tremble, worms believe that they are pursued by a mole, and leave their burrows. I beat the ground in many places where worms abounded, but not one emerged. When, however, the ground is dug with a fork and is violently disturbed beneath a worm, it will often crawl quickly out of its burrow."

Regarding smell, the interesting result was obtained, that the sense is "confined to the perception of certain odors"—namely, those emitted by natural food. For while the animals showed themselves indifferent to tobacco juice, paraffin, etc., held near them, pieces of cabbage leaf, onions, etc., when buried near an earth worm, were always discovered by the animal.

The presence of taste was proved by the fact that the worm showed a preference for some varieties of cabbage over others; but "of all their senses, that of touch, including in the term the perception of vibration, seems much the most highly developed."

Worms are omnivorous, dragging pieces of meat as well as leaves into their burrows for the purpose of eating them. They smear the leaves so drawn in with a secreted fluid. This fluid is alkaline, and acts both on the starch granules and on the protoplasmic contents of the cells; it thus resembles in nature the pancreatic secretion, and serves partly to digest the leaves before they are taken into the alimentary canal—so constituting the only case of extra-stomachal digestion hitherto recorded in an animal—its nearest analogy being perhaps that of the digestive fluid of Drosera or Dionœa, "for here animal matter is digested and converted into peptone not within a stomach, but on the surface of the leaves."

We now came to one of the most interesting chapters, which deals with the habit of dragging down leaves, etc., into the burrows; for here the experiment elicited some very remarkable evidence of action which is apparently intelligent. These experiments are thus led up to.

"Worms seize leaves and other objects, not only to serve as food, but for plugging up the mouths of their burrows; and this is one of their strongest instincts. Leaves and petioles of many kinds, some flower peduncles, often decayed twigs of trees, bits of paper, feathers, tufts of wool and horse-hairs are dragged into their burrows for this purpose. . . When worms cannot obtain leaves, petioles, sticks, etc., with which to plug up the mouths of their burrows, they often protect them by little heaps of stones; and such heaps of smooth rounded pebbles may frequently be seen on gravel walks. Here there can be no question about food. A lady, who was interested in the habits of worms, removed the little heaps of stones from the mouths of several burrows and cleared the surface of the ground for some inches all round. She went out on the following night with a lantern, and saw the worms with their tails fixed in their burrows, dragging the stones inward by the aid of their mouths, no doubt by suction. 'After two nights some of the holes had eight or nine small stones over them; after four nights one had about thirty, and another thirty-four stones.' One stone which had been dragged over the gravel walk to the mouth of a burrow weighed two ounces; and this proves how strong worms are."

The object of this plugging Mr. Darwin surmises to be that "of checking the free ingress of the lowest stratum of air when chilled by radiation at night."

Now, concerning the apparent intelligence displayed in these plugging operations, Mr. Darwin "observed carefully how worms dragged leaves into their burrows; whether by their tips or bases or middle parts. It seemed more especially desirable to do this in the case of plants not natives to our country; for although the habit of dragging leaves into their burrows is, undoubtedly, instinctive with worms, yet instinct could not tell them how to act in the case of leaves about which their progenitors knew nothing. If, moreover, worms acted solely through instinct or an unvarying inherited impulse, they would draw all kinds of leaves into their burrows in the same manner. If they have no such definite instinct, we might expect that chance would determine whether the tip, base, or middle was seized. If both these alternatives are excluded, intelligence alone is left; unless the worm in each case first tries many different methods, and follows that alone which proves possible or the most easy; but to act in this manner and to try different methods makes a near approach to intelligence."

A large number of experiments were therefore tried with leaves of various shapes, and both of endemic and exotic species. The results showed unequivocally that the part of the leaf which the worm seized for the purpose of dragging the whole into the burrow was not a matter of chance, but in an overwhelming majority of cases that part of a leaf was seized by the dragging of which the leaf would offer least resistance to being drawn into the burrow. Thus, for instance, "the basal margin of the blade in many kinds of leaves forms a large angle with the foot-stalk; and if such a leaf were drawn in by the foot-stalk, the basal margin would come abruptly into contact with the ground on each side of the burrow, and would render the drawing in of the leaf very difficult. Nevertheless, worms break through their habit of avoiding the foot-stalk, if this part offers them the most convenient means for drawing leaves into their burrows."

Again, in the case of pine leaves consisting of two needles joined to a common base, it is almost invariably by this base that the worm draws in the pair of leaves, and it is evident that, as the worm cannot lay hold of the two diverging points at the same time, this is the only part of the leaf by seizing which they would be able to drag the whole into their burrows. Mr. Darwin tried in some leaves tying or cementing the two diverging points together; but the worms still preferred the bases. Still further to test the hypothesis of chance, elongated triangles were cut out of paper and given to the worms instead of leaves. Here "it might certainly have been expected, supposing that worms seized hold of the triangles by chance, that a considerably larger proportion would have been dragged in by the basal than by the apical part;" while, inasmuch as the latter was in a literal sense the thin end of the wedge, it was the part which intelligent action would be most likely to choose. The results of many experiments with these paper triangles showed that " nearly three times as many were drawn in by the apex as by the base. . . . We may therefore conclude that the manner in which the triangles are drawn into the burrows is not a matter of chance, . . . and we may infer—improbable as is the inference—that worms are able by some means to judge which is the best end by which to draw triangles of paper into their burrows."

On the question of defining such action as intelligent or non-intelligent, Mr. Darwin refers to the criterion " that we can safely infer intelligence when we see an individual profiting by its own individual experience ; " and he adds that " if worms are able to judge, either before or after having drawn an object close to the mouths of their burrows, how best to drag it in they must acquire some notion of its general shape," and thus guide their actions by the result of individual experience.

Assuredly these observations are most interesting, and it would seem well worth while to try whether, by a series of lessons with similar triangles of paper, an individual worm could be taught to lay hold of the apex in a greater and greater proportional number of cases ; if so, there could no longer be any question as to the intelligent nature of the action.

The only other obsarvations with which we are acquainted pointing to the existence of intelligence in annelids are those of Sir E. Tennant (" Natural History of Ceylon," p. 481).

The remaining chapters of the book are occupied with the subject of its title, and in their course many quantitative results are given of the amount of mold which worms are able to cast up. Thus, for instance, a certain field was thickly covered with marl. Twenty-eight years afterward this layer of marl was found buried by mould to a depth varying between twelve and fourteen inches. Several other similar cases are given, the most interesting being that of a field which adjoins Mr. Darwin's own house. This was last plowed in 1841, then harrowed, and left to become pasture land. Then

" For several years it was clothed with an extremely scant vegetation, and was so thickly covered with small and large flints (some of them half as large as a child's head) that the field was always called by my sons ' the stony field.' When they ran down the slope the stones clattered together. I remember doubting whether I should live to see these larger flints covered with vegetable mould and turf. But the smaller stones disappeared before many years had elapsed, as did every one of the larger ones after a time; so that after thirty years (1871) a horse could gallop over the compact turf from one end of the field to the other, and not strike a single stone with his shoes. To any one who remembered the appearance of the field in 1842, the transformation was wonderful. This was certainly the work of the worms, for though castings were not frequent for several years, yet some were thrown up month after month, and these gradually increased in numbers as the pasture improved. In the year 1871 a trench was dug on the above slope, and the blades of grass were cut off close to the roots, so that the thickness of the turf and of the vegetable mould could be measured accurately. . . The average accumulation of the mould during the whole thirty years was only 0.083 inch per year; but the rate must have been much slower at first, and afterwards considerably quicker."

Numberless other corroborative cases are given, but we have no further space to enter into their details. Large stones are slowly undermined and sunk by worms, and woodcuts are given to illustrate actual measurements made by Mr. Darwin or his sons of the rate of sinking in particular cases. These measurements show that in the course of two or three centuries large blocks of stone (*e. g.* 67 by 39 by 15 inches) may become completely buried. Thus we are not surprised to learn that old pavements and low walls are subject to the same process, and many instances are given which have been observed by Mr. Darwin or his sons of the remains of Roman houses buried so far beneath the soil that the latter has been plowed for years without any one having suspected the presence of walls and pavements beneath. In some cases the thickness of the mould or soil above such remains was found to be twenty, thirty, and even forty inches.

The actual weight of worm castings thrown up in one year was calculated in one case to amount to 18.12 tons per acre.

Such being the work that worms are able by their gradual and cumulative action to accomplish, it becomes evident, as pointed out in Mr. Darwin's paper more than forty years ago, that worms must play an important part in the process of denudation. This topic is therefore treated at length, and it is shown that over and above the mechanical action already described, worms materially assist the process of denudation by the chemical actions incidental to digestion. For

"The combination of any acid with a base is much facilitated by agitation, as fresh surfaces are thus continually brought into contact. This will be thoroughly effected with the particles of stone and earth in the intestines of worms during the digestive process; and it should be remembered that the entire mass of the mould over every field, passes, in the course of a few years, through their alimentary canals. Moreover, as the old burrows slowly collapse, and as fresh castings are continually brought to the surface, the whole superficial layer of mould slowly revolves or circulates; and the friction of the particles one with another will rub off the finest films of disintegrated matter as soon as they are formed. Through these several means minute fragments of rocks of many kinds and mere particles in the soil will be continually exposed to chemical decomposition; and thus the amount of soil will tend to increase."

And,

"The several human acids, which appear, as we have just seen, to be generated within the bodies of worms during the digestive process, and their acid salts, play a highly important part, according to the recent observations of Mr. Julien, in the disintegration of various kinds of rocks."

Further,

"The trituration of small particles of stone in the gizzards of worms is of more importance under a geological point of view than may at first appear to be the case; for Mr. Sorby has clearly shown that the ordinary means of disintegration, namely, running water and the waves of the sea, act with less and less power on fragments of rock the smaller they are."

This assistance which worms lend to the process of denudation is of special importance in the case of flat or gently inclined surfaces, for here it is not improbably the chief agent at work. Castings thown up during or shortly before rain flow for a short distance down an inclined surface, and the finest earth is washed completely away. Again, during dry weather, the disintegrated castings roll as little pellets, and a strong wind blows all the castings, even on a level field, to leeward.

One other observation must be quoted, which, besides being of interest in itself, also has reference to the important subject of denudation:

"Little horizontal ledges, one above another, have been observed on steep grassy slopes in many parts of the world. Their formation has been attributed to animals traveling repeatedly along the slope in the same horizontal lines while grazing, and that they do thus move and use the ledges is certain; but Prof. Henslow (a most careful observer) told Sir J. D. Hooker that he was convinced that this was not the sole cause of their formation."

It is then shown that the initial cause of these ledges is the burrowing of earthworms. For,

"If the little embankments above the Corniche Road, which Dr. King saw in the act of formation by the accumulation of disintegrated and rolled worm castings, were to become confluent along horizontal lines, ledges would be formed. Each embankment would tend to extend laterally by the lateral extension of the arrested castings; and animals grazing on a steep slope would almost certainly make use of every prominence at nearly the same level, and would indent the turf between them; and such intermediate indentations would again arrest the castings."

Thus, on the whole, it will be seen how important an agency in nature Mr. Darwin has shown the action of worms to be, so that, in his own concluding words, "it may be doubted whether there are many other animals which have playcd so important a part in the history of the world as have these lowly organized creatures.—*Geo. J. Romanes in Nature.*

